# The fabrication of white light-emitting diodes using the n-ZnO/NiO/p-GaN heterojunction with enhanced luminescence

**DOI:** 10.1186/1556-276X-8-320

**Published:** 2013-07-13

**Authors:** Mazhar Ali Abbasi, Zafar Hussain Ibupoto, Mushtaque Hussain, Omer Nur, Magnus Willander

**Affiliations:** 1 Department of Science and Technology, Physical Electronics and Nanotechnology Division, Campus Norrköping, Linköping University, Norrkoping, 60174, Sweden

**Keywords:** White light-emitting diode, ZnO nanorods, Nanotubes, NiO buffer layer

## Abstract

Cheap and efficient white light-emitting diodes (LEDs) are of great interest due to the energy crisis all over the world. Herein, we have developed heterojunction LEDs based on the well-aligned ZnO nanorods and nanotubes on the p-type GaN with the insertion of the NiO buffer layer that showed enhancement in the light emission. Scanning electron microscopy have well demonstrated the arrays of the ZnO nanorods and the proper etching into the nanotubes. X-ray diffraction study describes the wurtzite crystal structure array of ZnO nanorods with the involvement of GaN at the (002) peak. The cathodoluminescence spectra represent strong and broad visible emission peaks compared to the UV emission and a weak peak at 425 nm which is originated from GaN. Electroluminescence study has shown highly improved luminescence response for the LEDs fabricated with NiO buffer layer compared to that without NiO layer. Introducing a sandwich-thin layer of NiO between the n-type ZnO and the p-type GaN will possibly block the injection of electrons from the ZnO to the GaN. Moreover, the presence of NiO buffer layer might create the confinement effect.

## Background

Zinc oxide (ZnO) is very much popular among the researchers due its wide direct band gap (3.37 eV) and high exciton binding energy (60 meV) at room temperature. The wide band gap and high exciton binding energy provides a solid platform for the ZnO in the fabrication of optoelectronic nanodevices. Specifically, light-emitting diodes (LEDs) and laser diodes based on the applications of the ZnO material explored its usability, thus ZnO-based light-emitting diodes are considered as the next-generation light-emitting diodes due to their cheap fabrication process and enhanced optical properties [1]. Several synthesis routes have been used for the fabrication of ZnO films and nanostructures, and the prepared ZnO material exhibits good crystalline and optical properties [2-4]. Recently, some ZnO p-n homojunction-based light-emitting diodes have been fabricated [5-7]. Due to the absence of a stable and reproducible p-type doped material with desired quality, ZnO-based light-emitting diodes are not considered up to the level of commercialization. Because of the lack of stable p-type ZnO, most ZnO heterojunctions are developed with the other existing p-type materials including p-type GaN [8-10], Si [9] and SiC (4H) [10]. Gallium nitride (GaN) is used effectively in the fabrication of heterojunction with ZnO for the development of light-emitting diodes because both materials exhibit a similar crystal wurtzite structure and electronic properties and differ by 1.8% lattice mismatch. The ZnO material is accompanied by the deep-level photoluminescence and electroluminescence (EL) in addition to near-band gap UV emission [11-14]. The deep-level emission is a critical issue which is not yet clear, but it is generally accepted that the possible oxygen vacancies or zinc interstitials are responsible for deep-level emissions [15]. The deep-level emission given by ZnO covers the wide range of visible spectrum, and theoretically, white emission can be obtained by hybridizing the deep-level emission of ZnO with the blue emission of GaN.

In order to improve the luminescence of ZnO-based light-emitting diodes, an interlayer of any other suitable material acting as a buffer medium is highly required for the significant improvement of the internal structure because the interlayer provides a stable charge environment during hole and electron injections in the light emitting part of the diode. Since the introduction of interlayers, such as TiO_2_, Ag, MoO_3_, WO_3_ or NiO interlayers, of different materials has improved the performance of polymer LEDs significantly, it has brought the change in the barriers for electrodes and also increases the hole injection which in result lowers the turn on and working voltage [16-20]. It is also reported that when a thin layer of NiO is deposited at the anode of ITO, then it has enhanced the optoelectronic working activity of double-sided emission devices using the thin-film-based heterojunction of p-NiO and n-ZnO materials [21]. ZnO-based white light-emitting diodes have also been fabricated on GaN substrate by our group previously [22,23].

Herein, we have developed n-ZnO/p-GaN heterojunctions with the presence and absence of a NiO buffer layer. The NiO buffer layer was deposited by the sol-gel method prior to the growth of the ZnO nanorods and nanotubes on GaN substrate. Four devices are prepared with ZnO nanorods and nanotubes on the GaN substrate: two with NiO buffer layer and the other two without. The devices were characterised by the X-ray diffraction (XRD), scanning electron microscopy (SEM), parameter analyser and the cathodoluminescence (CL) and EL techniques.

## Methods

Commercially available p-type GaN substrate was used in the development of the present p-n heterojunction. Prior to the growth of the n-type ZnO nanorods, a NiO buffer layer was deposited by the following sol-gel method. A sol-gel of nickel acetate was prepared in the 2-methoxyethanol having a concentration of 0.35 M, and di-ethanolamine was added dropwise under vigorous stirring at 60°C for 2 h by keeping the 1:1 molar ratio of nickel acetate and di-ethanolamine constant. After the synthesis of the sol-gel, cleaned GaN substrate was spin coated with the prepared sol-gel three to five times for the deposition of a thin NiO buffer layer; consequently, the substrate was annealed at 180°C for 20 min. After the annealing, the sample was left in the preheated oven for 4 h at 450°C in order to have a pure phase of NiO. After the deposition of the NiO buffer layer, the substrates were spin coated two to three times with a seed layer of zinc acetate for the growth of the ZnO nanorods and likewise annealed at 120°C for 20 min. Then, the annealed substrates containing the NiO buffer layer were dipped vertically in an equimolar 0.075 M precursor's solution of zinc nitrate hexahydrate and hexamethylenetetramine for 4 to 6 h at 90°C. After the growth of the ZnO nanorods, the nanotubes were obtained by chemical etching using 5 M potassium chloride solution at 85°C for 14 to 16 h.

After the growth of the ZnO nanorods and nanotubes with and without a NiO buffer layer, SEM was used to investigate the morphology of the prepared samples. The X-ray diffraction technique was used for the study of crystal quality and elemental composition analysis. The heterojunction analysis was performed using a parameter semiconductor analyser. CL and EL studies were carried out for the investigation of luminescence response of the prepared devices.

For the device fabrication, the bottom contacts are deposited by the evaporation of the 20-nm thickness of nickel and the 40-nm thickness of gold layers, respectively. Insulating layer of Shipley 1805 photoresist (Marlborough, MA, USA) was spin coated for the filling of vacant spaces between the nanorods, nanotubes and the growth-free surface of the GaN substrate. Reactive ion etching was used for exposing the top surface of the ZnO nanorods and nanotubes for the top contact of aluminium.

## Results and discussion

**Figure 1:**
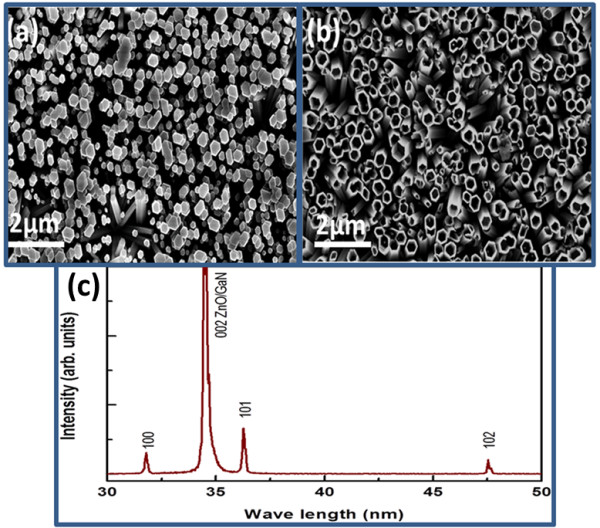
**SEM images and XRD pattern of ZnO. (a,b)** SEM images of as-grown ZnO nanorods and nanotubes on GaN. **(c)** XRD pattern of ZnO grown on GaN substrate.

Figure 1 represents the morphological study of both the ZnO nanorods and the nanotubes. The SEM image in Figure 1a has shown the uniform and well-aligned growth of nanorods. Also, almost all the nanorods are chemically etched as shown in Figure 1b. The X-ray diffraction study has shown good crystal quality with preferred *c*-axis orientation of the as-grown ZnO nanostructures. It can be seen that (002) crystal plane of ZnO seems more intense due to the similar X-ray diffraction pattern of GaN at (002) crystal plane as shown in Figure 1c.

**Figure 2:**
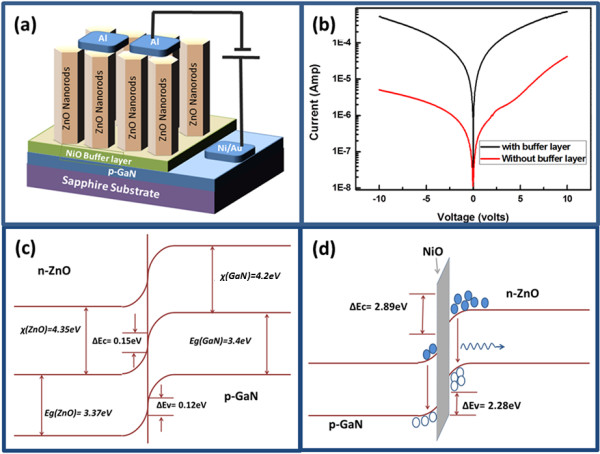
**Schematic diagram, I-V characteristic curves of proposed devices and band diagrams of p-n junctions. (a)** Schematic diagram of fabricated LED. **(b)** I-V characteristic curves of proposed devices based on ZnO nanorods with and without buffer layer of NiO. **(c,d)** Band diagrams of ZnO/GaN and ZnO/NiO/GaN p-n junctions, respectively.

The schematic diagram of the fabricated light-emitting diode based on the n-type ZnO/NiO/p-type GaN heterojunction is shown in Figure 2a. Figure 2b shows the I-V measurement of heterojunction diodes based on ZnO nanorods in the absence and presence of the NiO buffer layer. The I-V behaviour shown by both diodes is highly nonlinear and rectifying. It is also observed that the presence of the NiO buffer layer decreased the leakage current and showed higher series resistance compared to the device based on only n-ZnO/p-GaN heterojunction. Using the Au/Ni on p-type GaN and Al on n-type ZnO contacts has demonstrated acceptable Ohmic response, and it has also indicated that the rectifying response is solely coming from the n-type ZnO and p-type GaN heterojunction. With the help of Anderson's model, energy band diagram for the proposed devices is described using the band gap and the electron affinities of semiconducting materials. The band gaps of ZnO, NiO and GaN which have been taken from the reported work are 3.37, 3.86 [24] and 3.4 eV, respectively, while the electron affinities for ZnO, NiO and GaN are 4.35 [25], 1.46 [26] and 4.2 eV [27], respectively. Energy barrier for holes and electrons at the interfaces of the ZnO/NiO and the NiO/GaN are found to be 2.89 and 2.28 eV, respectively; the calculated values of electron and hole barriers for the n-ZnO/p-GaN are 0.15 and 0.12 eV, respectively as shown in Figure 2c,d. The difference of the energy band offsets in the presence of the NiO buffer layer is slightly higher than that without the NiO buffer layer. This indicates that the presence of the NiO buffer layer might block the transport of electrons from the ZnO to the GaN and also work as the hole injection source in the device. Also, the emission is more probably coming from the ZnO.

**Figure 3:**
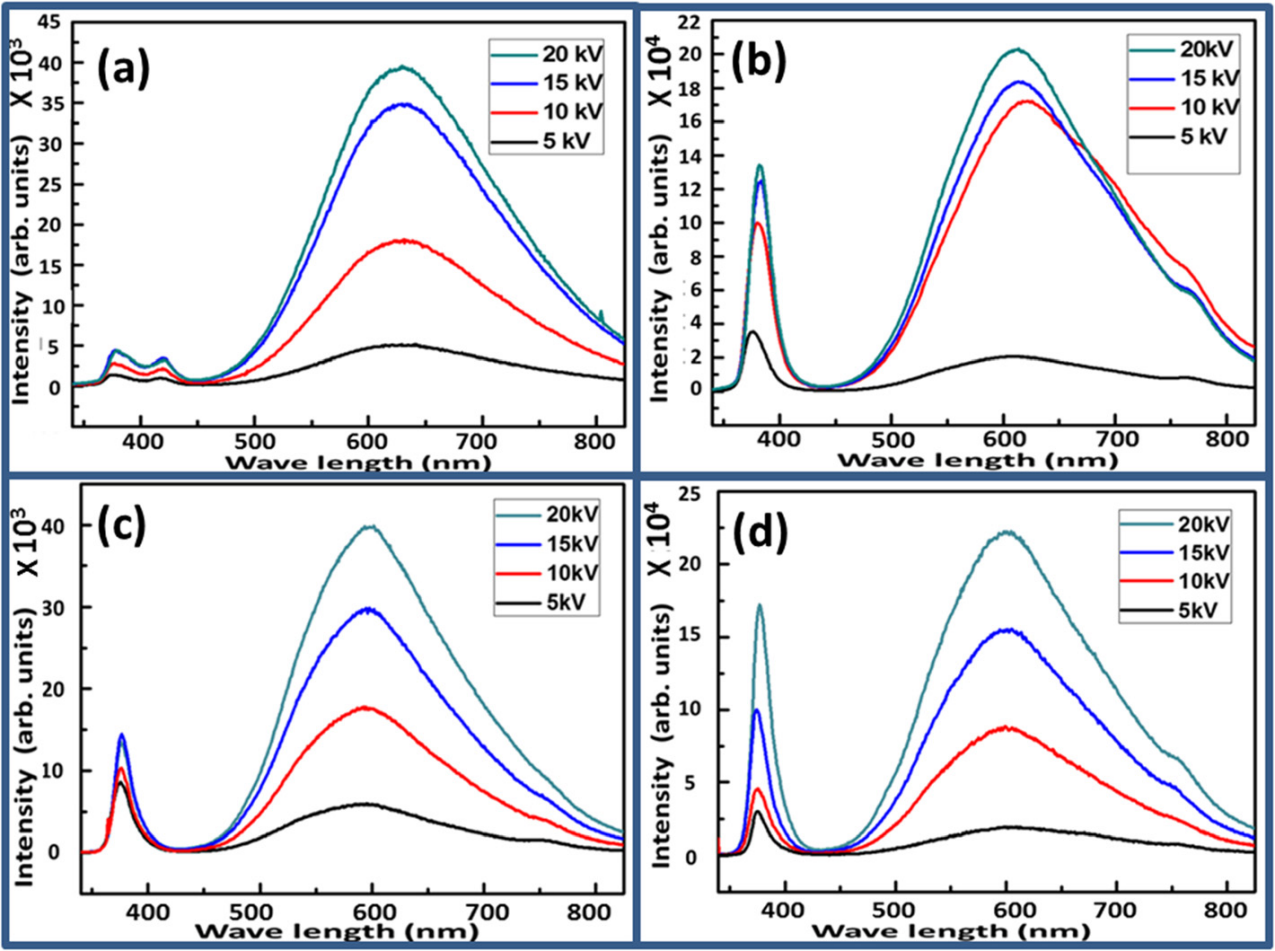
**CL spectra of nanorods and nanotubes without and with NiO buffer layer.** ZnO nanorods **(a)** on GaN and **(b)** on NiO thin-layer-coated GaN. ZnO nanotubes **(c)** on GaN and **(d)** on NiO thin-layer-coated on GaN.

Cathodoluminescence spectra have been recorded at room temperature, and the luminescence response of the fabricated n-type ZnO/p-type GaN heterojunctions with and without NiO buffer layer at different accelerating voltages is shown in Figure 3. Figure 3a,b shows room-temperature luminescence spectra for the ZnO-nanorod-based heterojunction without and with NiO buffer layer, respectively. It can be seen that a small peak at 425 nm is originating from the GaN substrate; however, a weak UV peak and a wide broad peak in the visible regions are also observed as shown in Figure 3a. Using the NiO buffer layer, the luminescence properties of the n-type ZnO nanorods/p-type GaN heterojunction are highly improved as shown in Figure 3b. The used NiO buffer layer has enhanced the luminescence properties due to more favourable hole injections and double recombination compared to the heterojunction without NiO buffer layer. It can be observed that the accelerating voltage has also made an influence on the local luminescence properties of the fabricated heterojunctions. The measured spectra showed that the number of excited carriers seems in proportion with the accelerating voltage. Similarly, ZnO-nanotube-based heterojunctions were developed without and with NiO buffer layer on the GaN substrate, and the luminescence behaviour was studied by the CL technique as shown in Figure 3c,d, respectively. It can be observed that the NiO buffer layer has also shown the same luminescence trend as in the case of the ZnO nanorods.

**Figure 4:**
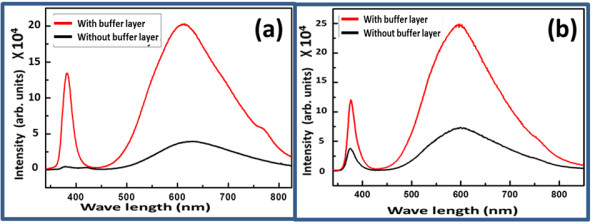
**Comparative CL spectra of ZnO nanorods and nanotubes with and without buffer layer. (a)** CL spectra of ZnO nanorods **(b)** CL spectra of ZnO nanotubes.

Figure 4 shows the CL spectra for the comparative study of nanorods and nanotubes based on devices at a fixed voltage of 20 kV. It can be clearly seen that the NiO has significantly contributed for the enhanced luminescent performance of the prepared light-emitting diodes compared to the light-emitting diode without a NiO buffer layer.

**Figure 5:**
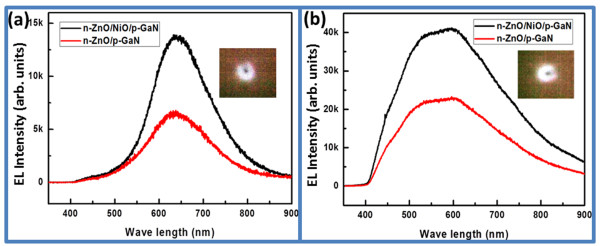
**EL spectrum of n-ZnO/p-GaN and n-ZnO/NiO/p-GaN. (a)** ZnO nanorods and **(b)** ZnO nanotubes. Insets show digital photographs of ZnO nanorod- and nanotube-based LEDs with NiO buffer layer.

The room temperature EL of the fabricated LEDs under forward bias at a constant current of 15 mA is shown in Figure 5. Figure 5a shows the EL response for the n-type ZnO nanorods/p-type GaN-developed LED in the presence and absence of the NiO buffer layer. In addition to the fabrication of NiO-buffer-layer-based LEDs with ZnO nanorods, the ZnO-nanotube-based LEDs were also produced. The EL spectra are shown in Figure 5b. It can be inferred that by introducing the NiO buffer layer, the luminescence properties of LEDs are significantly improved due to more injection holes, and a large number of electron-hole recombination is taking place at the interface. The wide broad green and orange-red emissions at 640 nm are strongly supported by the CL study for both LED devices based on ZnO nanorods and nanotubes using the NiO buffer layer. The presence of NiO buffer layer probably blocks the electron injection from the ZnO to the GaN because the smaller electron affinity (1.46 eV) and large band gap (3.86 eV) of NiO could have possibly raised the height of the conduction band barrier. Thus, the recombination of carriers is followed in the ZnO nanorods, and the luminescence is radically increased. Moreover, the insets of Figure 5a,b show the digital photographs of nanorod- and nanotube-based LEDs with a NiO buffer layer. The luminescence properties of the buffer-layer-containing LEDs are strongly enhanced compared to those without NiO buffer layer, ZnO nanorod- and nanotube-based LEDs; this can be attributed to more hole injections and a large number of electron-hole recombination at the interface.

## Conclusion

In this study, n-type ZnO/p-type GaN- and n-type ZnO/NiO/p-type GaN-based white light-emitting diodes are designed using two known morphologies of ZnO including nanorods and nanotubes. ZnO nanorods were well aligned and perpendicular to the GaN substrate, and some of the samples were almost fully chemically etched into nanotubes. XRD study shows the *c*-axis-oriented growth of the ZnO crystal structure with the possible involvement of GaN at (002) crystal plane. Both the CL and EL intensities were significantly increased by inserting a thin layer of NiO at the interface between the n-type ZnO and the p-type GaN due to possible blocking of electron injections from the ZnO to the GaN. Using the NiO buffer layer, the confinement is created which helps in the development of efficient LEDs based on n-type ZnO/NiO/p-type GaN heterojunctions.

## Competing interests

The authors declare that they have no competing interests.
